# Low skeletal muscle mass is associated with low aerobic capacity and increased mortality risk in patients with coronary heart disease – a CARE CR study

**DOI:** 10.1111/cpf.12539

**Published:** 2018-08-30

**Authors:** Simon Nichols, Alasdair F. O'Doherty, Claire Taylor, Andrew L. Clark, Sean Carroll, Lee Ingle

**Affiliations:** ^1^ Centre for Sports and Exercise Science Sheffield Hallam University Sheffield UK; ^2^ Department of Sport, Exercise and Rehabilitation Northumbria University Newcastle‐Upon‐Tyne UK; ^3^ Carnegie School of Sport Leeds Beckett University Leeds UK; ^4^ Academic Cardiology Castle Hill Hospital Cottingham UK; ^5^ Sport Health and Exercise Science University of Hull Hull UK

**Keywords:** cardiorespiratory fitness, coronary disease, sarcopenia, skeletal muscle

## Abstract

**Background:**

In patients with chronic heart failure, there is a positive linear relationship between skeletal muscle mass (SMM) and peak oxygen consumption ($\dot{V}$O_2peak_); an independent predictor of all‐cause mortality_._ We investigated the association between SMM and $\dot{V}$O_2peak_ in patients with coronary heart disease (CHD) without a diagnosis of heart failure.

**Methods:**

Male patients with CHD underwent maximal cardiopulmonary exercise testing and dual X‐ray absorptiometry assessment. $\dot{V}$O_2peak,_ the ventilatory anaerobic threshold and peak oxygen pulse were calculated. SMM was expressed as appendicular lean mass (lean mass in both arms and legs) and reported as skeletal muscle index (SMI; kg m^−2^), and as a proportion of total body mass (appendicular skeletal mass [ASM%]). Low SMM was defined as a SMI <7·26 kg m^−2^, or ASM% <25·72%. Five‐year all‐cause mortality risk was calculated using the Calibre 5‐year all‐cause mortality risk score.

**Results:**

Sixty patients were assessed. Thirteen (21·7%) had low SMM. SMI and ASM% correlated positively with $\dot{V}$O_2peak_ (*r* = 0·431 and 0·473, respectively; *P*<0·001 for both). SMI and ASM% predicted 16·3% and 12·9% of the variance in $\dot{V}$O_2peak_, respectively. SMI correlated most closely with peak oxygen pulse (*r* = 0·58; *P*<0·001). SMI predicted 40·3% of peak $\dot{V}$O_2_/HR variance. ASM% was inversely associated with 5‐year all‐cause mortality risk (*r* = −0·365; *P* = 0·006).

**Conclusion:**

Skeletal muscle mass was positively correlated with $\dot{V}$O_2peak_ in patients with CHD. Peak oxygen pulse had the strongest association with SMM. Low ASM% was associated with a higher risk of all‐cause mortality. The effects of exercise and nutritional strategies aimed at improving SMM and function in CHD patients should be investigated.

## Introduction

Peak oxygen uptake ($\dot{V}$O_2peak_), measured by a maximal cardiopulmonary exercise test (CPET) represents the upper limit of aerobic capacity. A low $\dot{V}$O_2peak_ is associated with the loss of independence in older individuals (Shephard, 2009) and increased all‐cause and cardiovascular mortality in patients with coronary heart disease (CHD) (Keteyian *et al*., 2008). The physiological factors that limit $\dot{V}$O_2peak_ are summarized by the Fick equation (Bassett & Howley, 2000; Poole *et al*., 2012; Lundby *et al*., 2017):

$$\dot{V}O_{2\mathrm{peak}}=\text{cardiac output}\;\times \;(a-{\text{vO}}_{2}\;\text{diff})$$

Where *cardiac output* is the product of heart rate (HR) and stroke volume (SV), and *a*−vO_2_ diff is the difference between arterial and venous O_2_ content, representing muscle O_2_ extraction. In healthy individuals, $\dot{V}$O_2peak_ is limited centrally; maximum cardiac output limits O_2_ delivery to the exercising muscle at the rate that it is required for aerobic resynthesis of ATP (Bassett & Howley, 2000; Lundby *et al*., 2017). However, in patients with chronic heart failure (CHF), a cascade of events alters peripheral muscle physiology. These include, reduced skeletal muscle oxidative enzyme activity, reduced mitochondrial density, decreased perfusion matching with oxidative muscle fibres (Poole *et al*., 2012) and decreased skeletal muscle mass (Cicoira *et al*., 2001). Consequently, the peripheral muscle may become the primary limitation to $\dot{V}$O_2peak_ (Clark *et al*., 1996; Shelton *et al*., 2010; Poole *et al*., 2012).

As CHF worsens, skeletal muscle mass (Collamati *et al*., 2016) and $\dot{V}$O_2peak_ decline (Piepoli *et al*., 2006; Fülster *et al*., 2013). However, this relationship has not yet been reported in patients with CHD. Around a quarter of patients with CHD have low muscle mass and function (sarcopenia) (Harada *et al*., 2017), compared with only 10% of adults older than 60 years (Shafiee *et al*., 2017). Patients with CHD are commonly physically inactive which, together with progression of the underlying CHD, may exacerbate loss of muscle mass. Patients with CHD who experience a marked loss of skeletal muscle mass may have a reduced $\dot{V}$O_2peak_ and consequently higher risk of early mortality. Identifying the relationship between skeletal muscle mass and $\dot{V}$O_2peak_ in patients with CHD may be important so that preventative exercise and nutritional interventions can be developed.

We aimed to describe the association between skeletal muscle mass and $\dot{V}$O_2peak_ in male patients with CHD. We also assessed the relationship between skeletal muscle mass and other potentially important variables: peak oxygen pulse (peak $\dot{V}$O_2_/HR) (Laukkanen *et al*., 2006), ventilatory anaerobic threshold (VAT) (Gitt *et al*., 2002), $\dot{V}$E/$\dot{V}$CO_2_ slope (Van de Veire *et al*., 2006), N‐terminal pro B‐type Natriuretic Peptide (NT‐proBNP) (Omland *et al*., 2007) and CALIBER 5‐year all‐cause mortality risk (Rapsomaniki *et al*., 2014).

## Methods

### Study design

Data for this cross‐sectional study included baseline measurements taken from male patients enrolled in the Cardiovascular and cardiorespiratory Adaptations to Routine Exercise‐based Cardiac Rehabilitation (CARE CR) study (Nichols *et al*., 2018). Ethical approval was obtained from the Humber Bridge NHS Research Ethics Committee – Yorkshire and the Humber (13/YH/0278). Study procedures conform to the declaration of Helsinki 1964.

The study protocol for CARE CR has previously been reported (Nichols *et al*., 2018). Briefly, clinically stable male patients who had recently been discharged from hospital following an admission for stable angina, myocardial infarction (MI), coronary artery bypass graft (CABG) surgery or elective percutaneous coronary intervention (PCI) were recruited. Patients were asked to attend the research laboratory having not participated in strenuous exercise within the previous 24 h. Written informed consent was obtained prior to conducting any investigations.

### Resting measurements

Resting HR and left arm brachial blood pressure were taken at the end of 15 min semi‐supine rest using a 12‐lead ECG (GE Healthcare, Buckinghamshire, UK) and an ECG‐gated automated blood pressure cuff (Tango, SunTech Medical, Eynsham, UK). Stature (cm) was measured using a Leicester Height Measure (SECA, Birmingham, UK). Waist circumference measurements were taken 1 cm above the iliac crest, and hip measurements were taken from the widest aspect of the buttocks. Both measurements were recorded in cm, and the ratio of the two was calculated to determine waist‐to‐hip circumference ratio (ACSM 2017).

A 2D echocardiogram was used to determine left ventricular (LV) function. LV ejection fraction (LVEF) was calculated using Simpson's method from measurements of end‐diastolic and end‐systolic volumes on apical four‐ chamber and two‐chamber 2D views, following the guidelines of Lang and colleagues (Lang *et al*., 2015). LV systolic dysfunction was diagnosed if LVEF was ≤45%.

### Body composition

Body composition was determined using dual X‐ray absorptometry [DEXA] (Lunar iDXA, 255 GE Healthcare). Total body mass (kg), lean body mass (kg) and total fat (%) were determined using the Lunar iDXA's integrated software. BMI (kg m^−2^) was calculated using DEXA‐derived total body mass. Appendicular lean mass (ALM; total lean mass in both arms and legs) was calculated (kg) and indexed to derive skeletal muscle index (SMI; measured in kg m^−2^). ALM was also reported as a percentage of total body mass (appendicular skeletal mass; ASM%). Low skeletal muscle mass was defined as an SMI of <7·26 kg m^−2^ as recommended by international consensus guidelines (Cruz‐Jentoft *et al*., 2010). A low ASM% was defined as <25·72% (Levine & Crimmins, 2012). This approach may be more appropriate for patients who are overweight/obese and have a higher absolute skeletal muscle mass, but low skeletal muscle mass relative to their total body mass.

### Maximal cardiopulmonary exercise test

The cardiopulmonary exercise testing adhered to established guidelines and recommendations (American Thoracic Society/American College of Chest Physicians 2003; Balady *et al*., 2010; Nichols *et al*., 2015; Taylor *et al*., 2015) and was performed using the modified Bruce treadmill protocol (Bruce *et al*., 1973) (GE Healthcare). A 12‐lead ECG was monitored continuously throughout the test. An ECG‐gated automated BP measurement was recorded at the start of the test and at the second minute of each test stage until the end of the test. Rating of perceived exertion (RPE) scores (6‐20) was recorded at peak exercise (Borg, 1982). Breath‐by‐breath metabolic gas exchange data were collected using an Oxycon Pro metabolic cart (Jaeger, Hoechburg, Germany). $\dot{V}$O_2peak_ was defined as the mean $\dot{V}$O_2_ (ml) over the last 30 s of the test. $\dot{V}$O_2peak_ was also adjusted for body mass (ml kg^−1^ min^−1^). The VAT was analysed by two independent investigators using the V‐slope method (Beaver *et al*., 1986) with data averaged over the middle five of seven consecutive breaths. The VAT was reported in ml, and standardized to patient body mass (ml kg^−1^ min^−1^). $\dot{V}$O_2_/HR (ml per beat) and $\dot{V}$E/ $\dot{V}$CO_2_ slope were calculated as previously described (Nichols *et al*., 2015, 2018).

### Pulse wave velocity

Pulse wave velocity (PWV) between the brachium and ankle was measured (Vascular Explorer, Enverdis GmbH, Düsseldorf, Germany) after 15 min of semi‐supine rest (torso elevated 45°) in a quiet temperature controlled room (21°C). A blood pressure cuff was placed proximally to the left cubital fossa (brachial artery) and another placed proximally to the medial malleolus. Photoplethysmographic sensors were placed on the patients left index finger and left hallux. Oscillations in the pulse waves alter the volume of the blood pressure cuff and are converted to a PWV. A shorter PWV (ms) indicates more severe arterial stiffness and/or worse peripheral vascular health. The reproducibility of PWV measurement at a single time point has been shown to be good (intraclass correlation 0·72–0·86) (Sutton‐Tyrrell *et al*., 2001).

### Blood samples

Resting venous blood samples were collected in ethylenediaminetetraacetic acid (EDTA), potassium oxalate and serum separating tubes (SST). EDTA and potassium oxalate tubes were spun in a refrigerated (4°C) centrifuge at 3000 revolutions per min, for 15 min immediately after the blood draw. Samples collected in SST tubes were allowed to clot for 30 min prior to being centrifuged under the same conditions. Haematocrit and haemoglobin concentrations, neutrophil and lymphocyte count, and NT‐proBNP were analysed using a registered National Health Service (NHS) pathology laboratory (Castle Hill Hospital, Hull). All samples not analysed on the day of collection were stored in a −80°C freezer. The ABX Pentra 400 biochemistry auto analyser (Horiba, Montpellier, France) was used to analyse serum plasma glucose, and high sensitivity C‐reactive protein (hs‐CRP) in duplicate. Calibration and quality controls were conducted in accordance with manufacturer's guidelines.

### Prognosis – Calibre 5‐year all‐cause mortality risk

Five‐year risk of all‐cause mortality was calculated for each patient using the comprehensive online (https://www.caliberresearch.org/model) Calibre 5‐year risk score (Rapsomaniki *et al*., 2014). The Calibre risk score has been developed in a population of 100 000 patients with CHD. The model has good calibration and discrimination in internal and external validation (C‐Index 0·811) for all‐cause mortality. Importantly, the model does not include any fitness measurements in its calculation. Five‐year risk of all‐cause mortality was reported as a percentage. The variables included in the Calibre score are shown in Table 1.

**Table 1:** Variables included in the CALIBER 5‐year risk score

Categorical variables	Continuous variables
Sex	Age
Belongs to most deprived quintile	Total cholesterol
CAD diagnosis and severity	HDL
Interventions (last 6 months)	Heart rate
Smoking status	Creatinine
Hypertension/BP lowering medication	White cell count
Diabetes	Haemoglobin
Heart failure	
Peripheral arterial disease	
Atrial fibrillation	
Stroke	
Chronic renal disease	
COPD	
Cancer	
Chronic liver disease	
Depression	
Anxiety	

CAD, Coronary Artery Disease; BP, Blood Pressure; COPD, Chronic Obstructive Pulmonary Disease; HDL, High‐density Lipoprotein.

### Statistical analysis

Statistical analysis was performed using SPSS version 22 (IBM, New York, NY, USA). The distribution of the data were assessed visually and using the Shapiro–Wilk test. Categorical data are reported as percentages. Continuous normally distributed variables are displayed as mean with 95% confidence intervals (95% CI) or standard deviation (±) where specified. Non‐normally distributed data are displayed as median (range). Pearson's (normally distributed), Spearman's correlations (non‐normally distributed) and age‐adjusted partial correlations were used to assess the relationship between indices of skeletal muscle mass and variables of interest. An *r* value of <0·25, 0·26–0·50, 0·51–0·75, and, >0·75 were considered weak, moderate, fair and strong associations, respectively (Berg & Latin, 2008). Scatter plots of partial correlations were constructed using the residuals of the independent skeletal muscle indices and dependent variables. Where a variable was significantly associated with SMI or ASM%, receiver operating characteristic (ROC) curves were used to investigate the sensitivity and specificity of predicting low skeletal muscle mass. Patients with a low skeletal muscle mass (SMI <7·26 Kg m^−2^ or ASM% <25·72%) were treated as the dichotomous ‘state‐variable’ for the ROC curve. Statistical significance was set at *P* = 0·05.

Up to six dependent variables with the strongest, significant age‐adjusted partial correlations were selected for inclusion within separate stepwise multivariate regression models. The main outcome variables selected were $\dot{V}$O_2peak_, VAT, peak $\dot{V}$O_2_/HR, CPET duration, Calibre 5‐year all‐cause mortality risk and NT‐proBNP.

## Results

Sixty male patients (aged, 62·1 ± 10·0 years; BMI, 28·8 ± 3·7 kg m^−2^) were recruited. Patient characteristics, comorbidities and medications are reported in Tables 2, 3 and 4, respectively. Fifteen patients (25·0%) had sustained a ST‐elevation MI, 19 (31·7%) a non‐ST‐elevation MI, 16 (26·7%) underwent elective PCI, 6 (10·0%) CABG and 4 (6·7%) had exertional angina. Comorbidities are shown in Table 3. Median time from cardiac event to baseline assessment was 54 days (range 22–220 days). Mean resting HR was 82 bpm ± 14 bpm. Mean resting systolic and diastolic blood pressure was 127 ± 17 mmHg and 58 ± 9 mmHg, respectively. Four patients (6·6%) had a LVEF <45%, ten (16·7%) had an NT‐proBNP >400 pg l^−1^ and one (1·7%) had an NT‐proBNP >2000 pg l^−1^. The proportion of patients with low skeletal muscle mass was 16·7% by SMI and 11·7% by ASM%. However, only four patients had both a low SMI *and* ASM%, meaning that 13 (21·7%) had a low SMI *or* ASM%.

**Table 2:** Patient characteristics

Variable	Mean (±SD)
Age (Years)	63·1 (10·0)
BMI (kg m^−2^)	28·8 (3·7)
ALM (kg)	24·8 (4·0)
SMI (kg m^−2^)	8·3 (1·1)
ASM%	29·1 (2·5)
Body fat%	35·5 (9·3)
Waist/Hip ratio	0·98 (0·06)
$\dot{V}$O_2peak_ (ml kg^−1^ min^−1^)	24·0 (5·6)
$\dot{V}$O_2peak_ (ml)	2079·3 (552·6)
$\dot{V}$O_2peak_ Lean (ml kg^−1^ min^−1^)	38·5 (8·3)
VAT (ml kg^−1^ min^−1^)	17·2 (5·1)
VAT (ml)	1481·9 (452·4)
VAT Lean (ml kg^−1^ min^−1^)	27·8 (7·6)
Peak $\dot{V}$O_2_/HR (ml per beat)	15·5 (3·3)
Peak HR (bpm)	134 (20)
$\dot{V}$E/$\dot{V}$CO_2_ slope	34·3 (6·0)
CPET duration (s)	826·6 (193·8)
LVEF (%)	54·3 (6·7)
Calibre 5‐year risk (%)	8·3 (7·0)
NT‐proBNP (pg l^−1^)	174·5 (11·4–2735·0)
hs‐CRP (mg l^−1^)	2·3 (3·0)
Glucose (mmol l^−1^)	6·1 (2·0)

BMI, body mass index; ALM, appendicular lean mass; SMI, skeletal muscle index; ASM%, appendicular skeletal mass; $\dot{V}$O_2peak_, peak oxygen uptake; VAT, ventilatory anaerobic threshold; $\dot{V}$O_2_/HR, oxygen pulse; HR, heart rate; bpm, bats per minute; $\dot{V}$E/$\dot{V}$CO_2_ slope, ventilatory efficiency with respect to carbon dioxide elimination; CPET, cardiopulmonary exercise test; LVEF, left ventricular ejection fraction; NT‐proBNP, N‐terminal pro B‐type natriuretic peptide; hs, high sensitivity.

**Table 3:** Patient comorbidities (Frequency)

Comorbidity	All patients
Hypertension (%)	27 (45·0)
Diabetes (%)	11 (18·3)
COPD (%)	2 (3·3)
Hyperlipidaemia (%)	38 (63·3)
Hypothyroidism	5 (9·1)
Previous PCI (%)	13 (21·7)
Previous MI (%)	14 (23·4)
Previous CABG (%)	4 (6·7)
Previous cardiac valve surgery (%)	1 (1·7)
Previous CVA (%)	6 (10·0)
Previous Cancer	8 (13·3)

COPD, chronic obstructive pulmonary disease; PCI, percutaneous coronary intervention; MI, myocardial infarction; CABG, coronary artery bypass graft; CVA, cerebrovascular accident.

**Table 4:** Patient medications (Frequency)

Medications	All patients
Aspirin (%)	58 (96·7)
Clopidogrel (%)	16 (26·7)
Ticagrelor (%)	33 (55·0)
Beta‐Blockers (%)	54 (90·0)
ACE Inhibitors (%)	38 (63·3)
Statins (%)	57 (95·0)
Diuretics (%)	5 (8·3)
Nitrates (%)	13 (21·7)
GTN (%)	54 (90·0)

ACE, angiotensin converting enzyme; GTN, glyceryl trinitrate.

**Figure 1:**
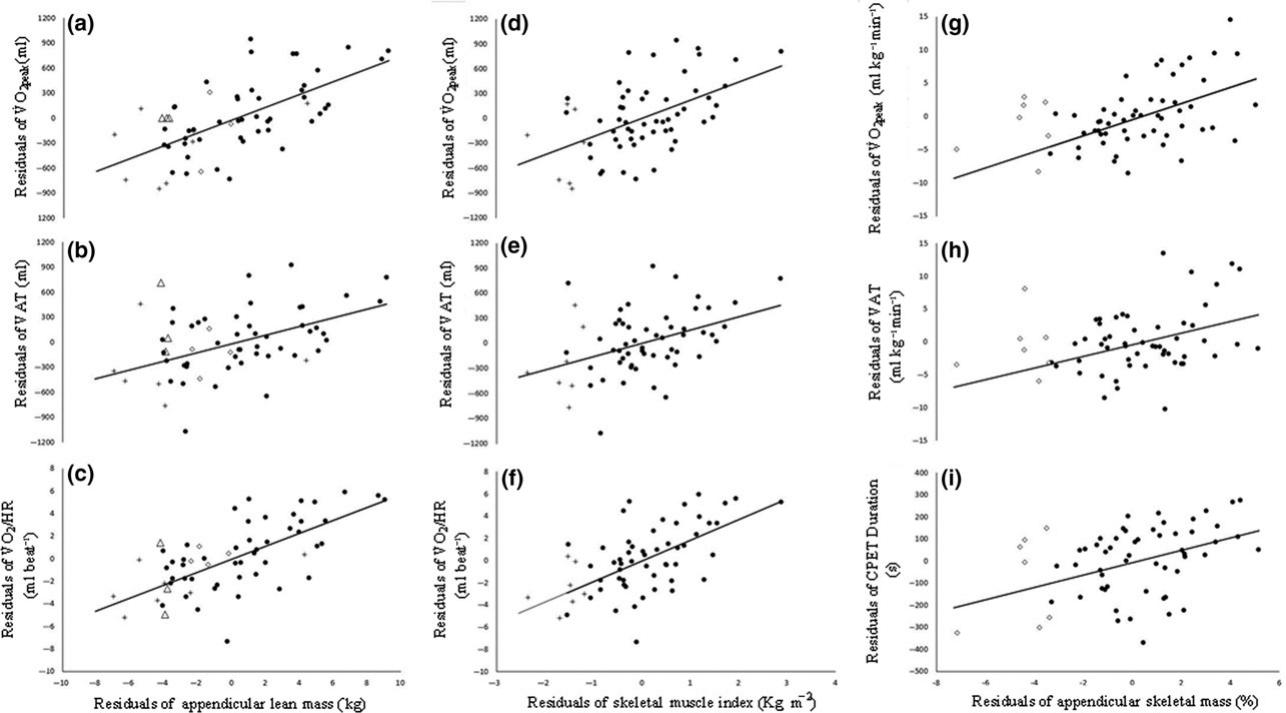
Partial correlations between appendicular lean mass and $\dot{V}$ O_2peak_ (a), VAT (b), and peak $\dot{V}$O_2_/HR (c). Panels d to f show partial correlations between skeletal muscle index and $\dot{V}$ O_2peak_ (d), VAT (e) and peak $\dot{V}$ O_2_/HR (f). Panels G to H show partial correlations between appendicular skeletal mass and $\dot{V}$ O_2peak_ (g), VAT (h), and total CPET Duration (i). $\dot{V}$ O_2peak_, peak oxygen uptake; VAT, ventilatory anaerobic threshold; $\dot{V}$ O_2_/HR, oxygen pulse; CPET, cardiopulmonary exercise test. * = Significant; + = Low muscle mass when defined using skeletal muscle index (<7·26Kg m^−2^); ◊ = Low muscle mass when defined using appendicular skeletal mass (<25·72%); ∆ = Low muscle mass when using either skeletal mass or appendicular skeletal mass.

Correlations between ALM, SMI, ASM% and dependent variables of interest are presented in Table 5. The associations between ALM, SMI and $\dot{V}$O_2peak_ (ml) were *r* = 0·566 (*P*<0·001) and *r* = 0·473 (*P*<0·001), respectively. The association between ASM% and $\dot{V}$O_2peak_ (ml kg^−1^ min^−1^) was *r* = 0·420 (*P* = 0·001). The strongest associations between indices of skeletal muscle mass and secondary outcome measures were observed between; ALM and peak $\dot{V}$O_2_/HR (*r* = 0·633; *P*<0·001), SMI $\dot{V}$O_2_/HR (*r* = 0·575; *P*<0·001) and ASM% and $\dot{V}$O_2peak_ [ml kg^−1^ min^−1^] (*r* = 0·431; *P*<0·001). CPET variables that were significantly associated with ALM, SMI and ASM% are shown in Fig. 1. ASM% was the only method of characterizing skeletal muscle mass resulting in a significant association with PWV, NT‐proBNP and Calibre 5‐year all‐cause mortality risk.

**Table 5:** Correlation and partial correlations between appendicular lean mass, skeletal muscle index, appendicular skeletal mass and dependent variables

Variable	Appendicular lean mass				Skeletal muscle index				Appendicular skeletal mass			
	Pearson's corr. (*r*)	*P*‐value	Partial corr. (*r*)	*P*‐value	Pearson's corr. (*r*)	*P*‐value	Partial corr. (*r*)	*P*‐value	Pearson's corr. (*r*)	*P*‐value	Partial corr. (*r*)	*P*‐value
$\dot{V}$O_2peak_ (ml)	0·666	0·001^a^	0·566	<0·001^a^	0·577	<0·001^a^	0·473	<0·001^a^	0·225	0·084	0·205	0·130
$\dot{V}$O_2peak_ (ml kg^−1^ min^−1^)	0·189	0·148	−0·007	0·962	0·196	0·134	0·042	0·758	0·420	<0·001^a^	0·431	0·001^a^
VAT (ml)	0·502	0·001^a^	0·360	0·006^a^	0·496	<0·001^a^	0·365	0·006^a^	0·167	0·203	0·148	0·277
VAT (ml kg^−1^ min^−1^)	0·053	0·686	−0·092	0·502	0·127	0·333	0·009	0·946	0·310	0·016^a^	0·312	0·019^a^
Peak $\dot{V}$O_2_/HR (ml per beat)	0·711	0·001^a^	0·633	<0·001^a^	0·643	<0·001^a^	0·575	<0·001^a^	0·202	0·121	0·163	0·230
Peak HR (bpm)	0·165	0·208	−0·042	0·752	0·110	0·402	−0·073	0·581	0·076	0·565	0·026	0·845
$\dot{V}$E/$\dot{V}$CO_2_ slope	−0·272	0·036^a^	−0·078	0·566	−0·270	0·037^a^	−0·099	0·468	−0·195	0·135	−0·158	0·245
CPET duration (s)	0·182	0·165	0·014	0·920	0·206	0·114	−0·047	0·730	0·376	0·003^a^	0·399	0·022^a^
LVEF (%)	0·153	0·244	0·077	0·574	0·137	0·297	0·068	0·620	0·076	0·566	0·055	0·698
Calibre 5‐year risk (%)	−0·426	0·001^a^	−0·230	0·870	−0·372	0·003^a^	−0·224	0·098	−0·330	0·010^a^	−0·365	0·006^a^
NT‐proBNP (pg l^−1^)^b^	−0·295	0·025^a^	0·155	0·254	−0·253	0·056	−0·131	0·337	−0·331	0·011^a^	−0·326	0·014^a^
hs‐CRP(mg l^−1^)	−0·092	0·486	−0·101	0·458	0·000	0·999	−0·004	0·974	−0·193	0·140	−0·184	0·175
Glucose (mmol l^−1^)	0·036	0·789	−0·051	0·708	0·135	0·314	0·065	0·634	−0·223	0·093	−0·242	0·073
Body fat (%)	0·149	0·254	0·153	0·261	0·151	0·249	0·148	0·275	−0·401	0·001^a^	−0·406	0·002^a^

corr., correlation; $\dot{V}$O_2peak_, peak oxygen uptake; VAT, ventilatory anaerobic threshold; $\dot{V}$O_2_/HR, oxygen pulse; HR, heart rate; bpm, beats per minute; $\dot{V}$E/$\dot{V}$CO_2_ slope, ventilatory efficiency with respect to carbon dioxide elimination; CPET, cardiopulmonary exercise test; LVEF, left ventricular ejection fraction; NT‐proBNP, N‐terminal pro B‐type natriuretic peptide; hs, high sensitivity.

a Significant.

b Spearman correlation.

### ROC curve analysis

Receiver operating characteristic curve analysis was conducted on variables that were significantly associated with measurements of SMI or ASM%. The area under the curve (AUC) for each prognostic variable is shown in Table 6. For SMI, peak $\dot{V}$O_2_/HR had the greatest predictive capacity (AUC = 0·767; *P* = 0·008). Values <13·3 ml per beat was predictive of a low SMI. When patients with a low SMI or ASM% were combined, peak $\dot{V}$O_2_/HR AUC was 0·764 (*P* = 0·004). A peak $\dot{V}$O_2_/HR <14·3 ml per beat was most predictive of patients with a low SMI or ASM%. Patients with a NT‐proBNP >112·5 pg l^−1^, a Calibre risk score >3·0% or a modified Bruce treadmill duration <17 min 43 s were also more likely to have a low ASM%.

**Table 6:** Area under the curve for variables associated low skeletal muscle mass (95% confidence intervals)

Variable	SMI		ASM%		SMI & ASM%	
	Area under the curve	*P*‐value	Area under the curve	*P*‐value	Area under the curve	*P*‐value
$\dot{V}$O_2peak_ (ml)	0·649 (0·478–0·820)	0·139	–	–	0·675 (0·527–0·823)	0·055
$\dot{V}$O_2peak_ (ml kg^−1 ^min^−1^)	–	–	0·726 (0·510–0·943)	0·053	0·615 (0·430–0·810)	0·206
Peak $\dot{V}$O_2_/HR (ml per beat)	0·767 (0·613–0·921)	0·008^a^	–	–	0·764 (0·632–0·895)	0·004^a^
VAT (ml)	0·594 (0·399–0·789)	0·351	–	–	0·620 (0·455–0·786)	0·187
VAT (ml kg^−1^ min^−1^)	–		0·679 (0·454–0·904)	0·126	0·561 (0·368–0·755)	0·501
CPET duration (s)	–		0·744 (0·510–0·943)	0·037^a^	0·648 (0·466–0·830)	0·104
Calibre 5‐year risk (%)	–		0·805 (0·600–1·00)	0·009^a^	0·383 (0·191–0·574	0·202
NT‐proBNP (pg l^−1^)	–		0·759 (0·511–1·00)	0·027^a^	0·623 (0·420–0·825)	0·178

SMI, skeletal muscle index; ASM%, appendicular skeletal mass; $\dot{V}$O_2peak_, peak oxygen uptake; $\dot{V}$O_2_/HR, oxygen pulse; VAT, ventilatory anaerobic threshold; CPET, cardiopulmonary exercise test; NT‐proBNP, N‐terminal pro B‐type natriuretic peptide.

a Significant.

### Multivariate regression

Appendicular lean mass and age (both *P*<0·001) were independent predictors of $\dot{V}$O_2peak_ (ml) and VAT (ml). For $\dot{V}$O_2peak_, these variables explained 58·8% of variance. For VAT 34·8% was explained. ALM alone accounted for 43·4% and 23·9% of variance for $\dot{V}$O_2peak_ and VAT, respectively. ALM was the only significant predictor of peak $\dot{V}$O_2_/HR (*P*<0·001), accounting for 49·8% of variance.

Skeletal muscle index and age were independent predictors of $\dot{V}$O_2peak_ and the VAT (ml). For $\dot{V}$O_2peak_, 52·5% of variance was explained by SMI and age, with SMI accounting for 16·3%. For VAT, 35·8% of variance was explained by SMI and age. About 12·1% of variance was explained by VAT alone. Interestingly, only SMI was an independent predictor for peak $\dot{V}$O_2_/HR. 40·3% of variance was accounted for by $\dot{V}$O_2_/HR.

Similar to SMI, ASM% and age (both *P*<0·001) were independent predictors of $\dot{V}$O_2peak_ and VAT, standardised to body mass (ml kg^−1^ min^−1^). 38·8% of $\dot{V}$O_2peak_ (ml kg^−1^ min^−1^) and 17·8% of VAT (ml kg^−1^ min^−1^) variance was accounted for by ASM% and age. ASM% alone accounted for 12·9% of $\dot{V}$O_2peak_ variance, compared to 6·3% for VAT. Age and ASM% were also independent predictors of NT‐proBNP (*P*<0·001) with age accounting for 20·5% of NT‐proBNP variance and ASM% accounting for 7·2% (combined model; 27·2%). ASM% accounted for 9·4% of total Calibre 5‐year all‐cause mortality risk score variance (*P*<0·001).

## Discussion

To our knowledge, this is the first study to investigate the relationship between skeletal muscle mass and $\dot{V}$O_2peak_ in patients with CHD. We identified that a lower skeletal muscle mass was associated with a lower $\dot{V}$O_2peak_, an observation previously reported in patients with CHF (Cicoira *et al*., 2001). We also investigated the relationship between other important prognostic indicators, including peak $\dot{V}$O_2_/HR. Our data suggest that skeletal muscle mass may be more closely associated with peak $\dot{V}$O_2_/HR, rather than $\dot{V}$O_2peak_. Similar to previously reported data in patients with CHD (Harada *et al*., 2017), we found that more than one‐fifth of patients had a low skeletal muscle mass; a higher proportion than reported among adults over the age of 60 years (10%) (Shafiee *et al*., 2017). A lower skeletal muscle mass was associated with a higher 5‐year all‐cause mortality risk.

### Peak oxygen uptake

Sarcopenia and/or a low muscle mass are associated with increased mortality risk (Cawthon *et al*., 2007) and difficulties performing daily activities (Janssen, 2006). A low $\dot{V}$O_2peak_ is also associated with a higher mortality risk (Keteyian *et al*., 2008) and difficulties performing daily activities (Shephard, 2009). Consistent with data reported on patients with CHF (*r* = 0·46 to *r* = 0·70) (Cicoira *et al*., 2001; Piepoli *et al*., 2006), we found that ALM was positively associated with $\dot{V}$O_2peak_ (*r* = 0·566). Although dependent on the methods used to scale $\dot{V}$O_2peak_, this relationship was maintained when ALM was standardized to stature (SMI; *r* = 0·473) and body mass (ASM%; *r* = 431). However, whilst ALM (43·4%), SMI (16·3%) and ASM% (12·9%) were independent predictors of $\dot{V}$O_2peak_, we were unable to replicate the same predictive strength (54‐65%) reported by Cicoira, *et al*. in non‐cachexic patients with CHF. This may be because patients with CHF can have severe skeletal muscle abnormalities (Clark *et al*., 1996; Vescovo *et al*., 1996; Piepoli *et al*., 2010; Poole *et al*., 2012) that limit exercise tolerance as a consequence of the disease (Shelton *et al*., 2010). Given that CHD represents an earlier stage of cardiovascular dysfunction than CHF, skeletal muscle abnormalities may occur at an earlier stage of cardiovascular dysfunction. Skeletal muscle mass may therefore play a greater role in limiting $\dot{V}$O_2peak_ among patients with CHF, compared with patients who have CHD. Our observation may suggest that adverse changes in skeletal muscle mass and quality associated with cardiovascular dysfunction exist on a continuum. If this were true, early optimization strategies that target peripheral muscle in addition to cardiac function may be important for disease prevention.

Peak $\dot{V}$O_2_/HR was most closely associated with indices of skeletal muscle mass. ALM and SMI separately accounted for 49·8% and 40·3% of the variance in peak $\dot{V}$O_2_/HR, respectively. These indices of skeletal muscle mass not only had a stronger association with peak $\dot{V}$O_2_/HR (*r* = 0·575 to *r* = 0·633) compared to $\dot{V}$O_2peak_ (*r* = 0·566), but also had the largest AUC (0·767; 95% CI 0·613–0·921; *P* = 0·008). Unlike $\dot{V}$O_2peak,_ peak $\dot{V}$O_2_/HR had good sensitivity and specificity for detecting patients with a low SMI. A threshold of 13·3 ml per beat was identified as the point below which, patients were more likely to have a low SMI (7·26 kg m^−2^). Simple rearrangement of the Fick equation means that peak $\dot{V}$O_2_/HR becomes independent of HR; a factor that we (*r* = −0·042, −0·073, 0·026; *P*>0·05 for all) and others (Horwich *et al*., 2009; Piepoli *et al*., 2017) have found to be unrelated to skeletal muscle. Instead, reported peak $\dot{V}$O_2_/HR characterizes SV and a‐vO_2_ difference (peripheral O_2_ extraction) which may explain why it appears to be more closely related to skeletal muscle mass than $\dot{V}$O_2peak_, in patients with CHD. However, whilst skeletal muscle mass has previously been identified as an independent predictor of peak $\dot{V}$O_2_/HR (74%) in hypertensive men and women (Lim *et al*., 2005), the association between peak indices of skeletal muscle mass and peak $\dot{V}$O_2_/HR in our study was smaller [40·3–49·8%] (Lim *et al*., 2005). Nonetheless, this is an interesting finding and should be further explored in a larger cohort.

The weaker relationship between indices of skeletal muscle mass and peak $\dot{V}$O_2_/HR observed in our study may indicate that patients with CHD have a different physiological response to maximal exercise, compared to patients with hypertension. Under normal circumstances, $\dot{V}$O_2_/HR rises progressively throughout an incremental exercise test until it reaches a plateau associated with normal physiological limitation to exercise (Whipp *et al*., 1996). However, in patients with CHD, $\dot{V}$O_2_/HR may prematurely decrease during incremental exercise due to ischaemia‐induced myocardial wall‐motion abnormalities which cause a reduction in SV (Belardinelli *et al*., 2003). This is thought to occur prior to ST‐segment changes detected using electrocardiogram, or symptoms of angina (Nesto & Kowalchuk, 1987). If myocardial blood flow is not restored through surgical or medical intervention, $\dot{V}$O_2_/HR would be lower than expected at peak exercise and may attenuate the association between $\dot{V}$O_2_/HR, a‐vO_2_ difference and skeletal muscle mass. Alternatively, a low peak $\dot{V}$O_2_/HR may indicate greater cardiac dysfunction and consequently, more severe skeletal muscle abnormalities. However, whilst we cannot confirm either of these scenarios, peak $\dot{V}$O_2_/HR was a better predictor of SMI than any other measured variable, including $\dot{V}$O_2peak,_ the gold‐standard measurement of aerobic exercise capacity. Formal screening for sarcopenia in patients who are incidentally found to have a low peak $\dot{V}$O_2_/HR may be beneficial.

### Prognostically important associations with skeletal muscle mass

Although SMI was associated with peak $\dot{V}$O_2_/HR, ASM% was not. However, ASM% was inversely associated with prognostically important variables including; $\dot{V}$O_2peak,_ PWV, 5‐year all‐cause mortality risk, a higher NT‐proBNP and, body fat percentage even when controlling for age. Although not specifically investigated by our study, the faster PWV speeds observed among patients with a lower SMI indicates more severe arterial stiffness and greater abnormalities in peripheral cardiovascular health, something reported among patients with CHF (Poole *et al*., 2012). Furthermore, the higher NT‐proBNP values reported among patients with a lower ASM% suggests more advanced CHD, or the early development of CHF which may exacerbate the loss of skeletal muscle mass (Collamati *et al*., 2016). The association between higher body fat percentage and relative lower skeletal muscle mass may also indicate the onset of sarcopenic obesity, fat infiltration of skeletal muscle and therefore reduced muscle quality (Narici & Maffulli, 2010).

### Strengths and weaknesses

This is the first study to use gold‐standard measurement techniques (DXA and CPET) to investigate the relationship between low skeletal muscle mass and reduced aerobic exercise capacity in CHD patients. In addition, this study included a representative cohort of male patients with CHD and the statistical methods employed controlled for several variables that are commonly associated with reduced skeletal muscle mass and aerobic exercise capacity. However, this study has limitations; first, we did not assess muscle function, which meant that we could not report the prevalence of sarcopenia in our cohort. Second, our findings in our male only cohort may not be relevant to females with CHD. In addition, the sample size used in this population was relatively small. Finally, whilst the associations identified in our cross‐sectional cohort study are interesting, a prospective long‐term follow‐up study is required. This would help to determine whether progression of CHD confers with reduced skeletal muscle mass and whether this relates to prognostically important variables and/or the development of CHF.

## Conclusion

We found a high incidence of low skeletal muscle mass in our cohort of patients with CHD. When standardized to body mass (ASM%), low skeletal muscle mass conferred a higher predicted risk of all‐cause mortality. Low skeletal muscle mass was associated with a low $\dot{V}$O_2peak_ in patients with CHD; however, the relationship was complex and dependent on the method used to scale skeletal muscle mass and $\dot{V}$O_2peak_. Interestingly, our data show that there was a stronger association between SMI and peak $\dot{V}$O_2_/HR. The relationship between low skeletal muscle mass, prognostic indices and aerobic fitness suggests that adverse changes in skeletal muscle mass may be initiated before the diagnosis or development of CHF. These findings may highlight a need for preventative exercise and nutritional strategies to improve skeletal muscle mass and quality in patients with CHD.

## Funding

Financial support for blood sample analysis was provided by the Hull and East Riding Cardiac Trust Fund (Hull, East Yorkshire, UK). Funding for a research post (SN) was supported by City Health Care Partnership CIC (Hull, UK). [No Grant Numbers Issued].

## Conflict of interest

SN received salary match‐funding from City Health Care Partnership CIC (Hull, UK) during the data collection period of this study. The authors declare no other conflict of interest.

## Author contributions

SN contributed to project conception and design, acquisition, analysis and interpretation of data, drafted and critically revised the manuscript. AFO'D contributed to analysis and interpretation of data, and drafted and critically revised the manuscript.CT contributed to the acquisition of data and critically revised the manuscript. ALC contributed to project design and critically revised the manuscript. SC and LI contributed to project conception and design, analysis and interpretation of data, drafted and critically revised the manuscript. All authors have given their final approval and agree to be accountable for all aspects of work ensuring integrity and accuracy.

## Manuscript status

We confirm that this manuscript is not under review with any other Journal. This manuscript has not previously been submitted or reviewed by another Journal. Findings reported in this manuscript have not been presented at any conference.
